# Discussion about the Latest Findings on the Possible Relation between Air Particulate Matter and COVID-19

**DOI:** 10.3390/ijerph20065132

**Published:** 2023-03-14

**Authors:** Maria Cristina Collivignarelli, Stefano Bellazzi, Francesca Maria Caccamo, Marco Carnevale Miino

**Affiliations:** 1 Department of Civil Engineering and Architecture, University of Pavia, Via Ferrata 3, 27100 Pavia, Italy; 2 Interdepartmental Centre for Water Research, University of Pavia, Via Ferrata 3, 27100 Pavia, Italy

**Keywords:** PM_10_, PM_2.5_, SARS-CoV-2, virus vector, coronavirus, air pollution, traffic, COVID-19

## Abstract

Since SARS-CoV-2 was identified, the scientific community has tried to understand the variables that can influence its spread. Several studies have already highlighted a possible link between particulate matter (PM) and COVID-19. This work is a brief discussion about the latest findings on this topic, highlighting the gaps in the current results and possible tips for future studies. Based on the literature outcomes, PM is suspected to play a double role in COVID-19: a chronic and an acute one. The chronic role is related to the possible influence of long-term and short-term exposure to high concentrations of PM in developing severe forms of COVID-19, including death. The acute role is linked to the possible carrier function of PM in SARS-CoV-2. The scientific community seems sure that the inflammatory effect on the respiratory system of short-term exposure to a high concentration of PM, and other additional negative effects on human health in cases of longer exposure, increases the risk of developing a more severe form of COVID-19 in cases of contagion. On the contrary, the results regarding PM acting as a carrier of SARS-CoV-2 are more conflicting, especially regarding the possible inactivation of the virus in the environment, and no final explanation on the possible acute role of PM in the spread of COVID-19 can be inferred.

## 1. Introduction

In December 2019, the first cases of COVID-19 were identified. Weeks later, the disease due to SARS-CoV-2 had expanded to many other countries and was declared as a pandemic by the World Health Organization at the beginning of March 2020 [1,2].

To date (February 2023), more than 750 million cases have been confirmed, and more than 6.5 million deaths have been ascertained [3]. Therefore, infections from SARS-CoV-2 have posed a severe risk to world health. While in the beginning, it was identified as a cause of fever, dry cough, headache, fatigue, and gastrointestinal symptoms, later studies have also demonstrated the onset of other disorders, such as acute cardiac injury [4,5].

In parallel with studies about the effects of SARS-CoV-2 on human health and possible mechanisms of action [6,7,8,9,10], the scientific community has started to investigate the link between particulate matter (PM) and the virus.

Several studies have proven that measures imposed against virus proliferation (e.g., partial and/or total lockdowns) limited travel and displacement, reducing the amount of PM in the air mainly due to the low traffic [11,12,13]. The scientific community has also pointed out the possible role of air particulate in the spread of COVID-19, investigating if PM could enhance the risk of developing a severe form of the disease and act as a carrier promoting the spread of the virus [14,15,16].

Previous studies have already determined a relationship between the concentrations of fine PM and the incidences of several acute respiratory infections due to viruses such as adenovirus, human coronavirus, and influenza virus. The results of one study highlighted a positive correlation with the co-influence of other environmental factors, such as temperature and humidity [17]. Another study suggested that exposure to a high concentration of PM can also compromise the immune system, altering the ability of alveolar macrophages to take up viruses, such as respiratory syncytial virus [18].

The possible airborne transmission of viruses and bacteria on particulate surfaces is already known. For instance, Zhao et al. [19] pointed out that the majority of people affected by avian influenza in the 2015 outbreak in Iowa (U.S.A.) might have been infected by the virus carried by PM from infected farms.

After the COVID-19 outbreak, the scientific community has tried to explain the relationship between PM and SARS-CoV-2, evaluating if it differs or not from its relationship with other viruses. Based on the latest literature findings, this work discusses the possible roles of PM in the spread of SARS-CoV-2, highlighting the gaps in the research and suggesting possible further steps of investigation.

Given the importance of enhancing knowledge about the relationship between viruses and environmental factors, this work provides indications useful to the scientific community and technicians of the sanitary and medical sectors. This information will be helpful not only to prevent/limit the spread of SARS-CoV-2 but also to face the challenges of other diseases in the future.

## 2. Method and Approach

This study presents a discussion about the latest findings about the possible role of PM in SARS-CoV-2 spread based on the latest published work. Given the aim of the work, the literature was searched on Scopus^®^ and Google Scholar using the keywords “Particular matter AND COVID-19” and “Particulate matter AND SARS-CoV-2”. As a criterion of exclusion, only works written in English and subjected to peer review before publication were considered. The works were then screened to avoid presenting papers not completely on the topic.

This work is divided into two main sections: one reporting the main findings about the relationship between COVID-19 and PM (Section 3), and the other presenting a short discussion mainly about the current gaps and tips for future studies (Section 4).

## 3. Roles of Particulate Matter in SARS-CoV-2 Spread

Regarding PM, the literature suggests two possible mechanisms of action in SARS-CoV-2 spread: a chronic and an acute role.

The chronic role is related to the effect on the population, especially in urban environments, of long-term and short-term exposure to PM at high concentrations. Studies have investigated if this condition could enhance the risk of developing more severe forms of COVID-19 with respect to unexposed populations.

The acute role is the action of PM as a carrier for SARS-CoV-2, promoting the spread of the virus.

### 3.1. Chronic Role of Particulate Matter

The negative effects on human health of chronic exposure to high concentrations of PM have been already determined by several studies. These negative effects are mainly due to the constituents of PM, such as nitrates, sulfates, organic compounds, bacteria and viruses, and heavy metals [20]. Moreover, the small size of the particles (<10 µm in the case of PM_10_, and <2.5 µm in the case of PM_2.5_) facilitates their inhalation by the respiratory system [21].

In addition to the respiratory system, which can be damaged (e.g., asthma attacks and chronic bronchitis) by short-term exposure to PM, long-term exposure can also cause other health dysfunction, such as diabetes, cardiovascular diseases, cancer, and premature deaths [20,22,23]. Davidson et al. [24] reported that, based on previous studies, an increase in PM_2.5_ is associated with an increase in the death rate due to many causes, such as respiratory problems, cardiovascular issues, and lung cancer.

After the spread of COVID-19, the scientific community started to investigate if PM can also play a chronic role that affects the impact of SARS-CoV-2 on human health. Prinz and Richter [25] studied how long-term exposure to fine particulate matter air pollution influences infections and deaths from COVID-19 in Germany. They collected the data on PM_2.5_ in more than 400 counties from the previous 19 years, correlating them with COVID-19 cases and fatalities. Their results highlighted substantial positive correlations of both with long-term particulate matter concentration exposure [25].

Bianconi et al. [26] focused on the link between PM and COVID-19 effects in more than 100 provinces in Italy. Their results showed that exposure to both fine and coarse PM was related to infections and deaths in the first wave (1–31 March 2020) [26].

In another study, Solimini et al. [27] investigated the global association between infections and PM at a regional scale. They collected and compared epidemiological data in the first COVID-19 wave at the beginning of 2020 with air quality data from 2014 to 2018. Additionally, other variables, such as demography and climate, were considered. Their study highlighted that increases of 10 μg m^−3^ in PM_2.5_ and PM_10_, linked with increases in pollution levels, were associated with increases of 5.4–10.5% and 7.8–14.9% in the number of infections, respectively [27].

Short-term exposure can also influence the effect of the disease due to SARS-CoV-2. For instance, in the United States, Zhou et al. [28] studied a possible correlation between an excess of COVID-19 cases and deaths and exposure to PM_2.5_ due to the 2020 wildfires, finding clear evidence that short-term exposure to PM at high concentrations amplified the impact of the disease.

Based on the literature, it is quite clear that exposure to PM at high concentrations can negatively affect the respiratory and cardiovascular system, leaving exposed people more susceptible to worse COVID-19 symptoms and death. However, other studies on the possible pattern of the co-presence of other air pollutants have been carried out, with some contrasting results. For instance, Travaglio et al. [29] compared the cases and deaths from SARS-CoV-2 infections with long-term air pollution exposure in England. They highlighted that PM_2.5_ was a major contributor to COVID-19 cases, also showing a positive correlation with other pollutants, such as NO_2_ [29]. Furthermore, Zang et al. [30] found a detrimental effect of the main air pollutants on the effects of COVID-19 on the exposed population.

The higher number of infections found in case of high PM concentrations could be related to a higher number of swab tests due to lower asymptomatic cases, but this aspect should also be further studied.

### 3.2. Acute Role of Particulate Matter

Several authors have already proven that PM can act as a carrier of pathogenic bacteria and viruses [31,32]. In general, pathogens can be adsorbed to PM particles through coagulation and stay in the air for up to weeks, reaching long distances [33]. After the COVID-19 outbreak, studies have tried to understand if SARS-CoV-2 can also be transported by air particulate and influence the spread of the disease. Many studies have investigated the presence of SARS-CoV-2 in PM during the different peaks of COVID-19.

Setti et al. [34] collected more than thirty samples in Bergamo (northern Italy) to research the presence of SARS-CoV-2 RNA in PM_10_ with three specific marker genes (E, N, and RdRP). They highlighted that 20 samples were positive for at least one marker gene, suggesting that PM can serve as a carrier for SARS-CoV-2 [34]. Nor et al. [35] focused on the role of PM_2.5_, collecting samples over four weeks in four separate indoor hospital environments. Although the study did not find a correlation between the concentration of PM_2.5_ and SARS-CoV-2, their results indicated that the air particulate was a possible carrier for the virus [35].

However, as often happens in frontier research, the results in the literature are conflicting. For instance, Pivato et al. [36] researched the presence of SARS-CoV-2 RNA with RT-qPCR in more than forty samples of PM_2.5_ and PM_10_ that were collected during the peak of the first wave in northern Italy. The results did not highlight the presence of the virus, showing that spread due to PM can be considered negligible [36]. Belosi et al. [37] reached a similar result, estimating the outdoor concentrations of SARS-CoV-2 in northern Italy through a simple box model approach. Even assuming a very high number of infections (25%), the results indicated a very low virus concentration (<1 SARS-CoV-2 RNA copy m^−3^) in the outdoor environment [37].

In any case, the presence of the virus RNA in PM is not necessarily linked to a higher spread of the disease. In fact, SARS-CoV-2 can be present but partially or completely inactivated due to external factors, such as the temperature and solar radiation, which can accelerate the inactivation rate [33].

Jarvis [38] focused on the factors that affect SARS-CoV-2 inactivation in aerosols. He highlighted that SARS-CoV-2 appeared to be less influenced by humidity with respect to other viruses but very sensitive to temperature and UVC radiation. Although its half-life in aerosol is generally higher than other coronaviruses, the presence of solar radiation generally reduces the half-life of SARS-CoV-2 in aerosols to 2–3 min [38].

These results were also confirmed by our previous study, in which the data on the PM concentrations in more than forty cities were collected and compared with the epidemic rapidity of COVID-19 spread (doubling time). Although the study did not take into consideration synergistic action with other factors (e.g., meteorological ones), the results seemed to exclude PM as a primary cause of the low COVID-19 doubling time in some areas of Italy [14].

De la Fuente et al. [39] evaluated the response of SARS-CoV-2 on PM surfaces and found that the virus was completely inactivated by interactions with engine exhaust PM, while it maintained a limited replication capacity when present on PM from other sources.

Regarding the possible interaction between SARS-CoV-2 and PM, Borisova and Komisarenko [40] pointed out that it happens in surrounding water. They also highlighted that PM and SARS-CoV-2 have the same nose-to-brain delivery mechanism and can bring the same interference of neuronal effects, aggravating each other’s symptoms [40].

## 4. Discussion, Current Gaps, and Tips for Future Studies

After the COVID-19 outbreak, the scientific community started to investigate the reason for the heterogeneous behavior of the contagion at a regional scale. Some authors pointed out that environmental factors, such as climate conditions, and air pollution could be directly linked to the diverse rapidity with which SARS-CoV-2 spread among the regions.

Regarding air pollutants, PM was one of the most investigated due to its well-known negative effects on human health. The main hypotheses of how it can influence the epidemic of COVID-19 can be summarized in two points: (i) a chronic effect of long-term and short-term exposure to high concentrations of PM, and (ii) an acute role of the particulate as a carrier of the virus.

### 4.1. Chronic Role of Particulate Matter

Regarding the chronic role of PM, all of the literature reviewed in the present work agrees about the negative impact of both long-term and short-term exposure to PM on the number of COVID-19 infections and their severity. The higher and longer the exposure, the higher the risk to contract the infection and show severe symptoms of the disease, including death. Severe symptoms could be related to the inflammatory status of the lungs and respiratory system in people previously exposed to significant concentrations of PM [41]. Moreover, exposure to fine particulate (PM_2.5_) could increase the incidence and severity of COVID-19, altering the expression of angiotensin-converting enzyme 2 (ACE2) in respiratory cells, which acts also as a receptor for SARS-CoV-2 [42]. In fact, a study proved that in mice, exposure to PM_2.5_ can increase the number of ACE2 receptors [43].

In addition, more severe symptoms reduced the number of asymptomatic cases, inducing a larger use of swab testing. This aspect could be the reason for a higher rate of infections in cases of chronic exposure to high concentrations of PM. 

Despite some contrasting results, a pattern between chronic exposure to PM and COVID-19 aggressiveness seems to be plausible. What it is not completely understood, and therefore, deserves further study, is the combined effect with variables such as other air pollutants, climate conditions, and socio-demographical aspects. In this case, multifactorial analysis is strongly suggested in future studies. This aspect is very important not only in relation to SARS-CoV-2 infections but also in cases of other hypothetical future pandemics. Completely understanding the environmental variables that can play a key role in the aggressiveness of airborne-transmitted viruses represents a fundamental step toward managing them in cases of new outbreaks.

### 4.2. Acute Role of Particulate Matter

People infected with SARS-CoV-2 can generate aerosols and droplets containing virus particles by coughing, sneezing, and talking. Depending on the size of the particles emitted (<5 μm or >5 μm), they are called aerosols or droplets, respectively. Among these, only particles with a lower diameter can generally travel for longer distances [44]. Preliminary studies have suggested the possibility that PM can act as a carrier for SARS-CoV-2, released by infected people as aerosols, as for other bacteria and viruses. However, the results on this point are conflicting, with several studies proving the presence of the virus attached to PM, while others demonstrating the absence of the virus RNA. The main reason for this discrepancy is probably due to the high influence of environmental conditions, such as temperature and solar radiation, on the inactivation and degradation of SARS-CoV-2. Considering that many variables influence the presence of the virus in PM, it is not simple to compare the data obtained in the studies, which have adopted different approaches for the sampling and detection of the virus.

Assuming that PM can effectively act as a carrier for SARS-CoV-2, this is not enough to prove a direct role of particulate in the promotion of COVID-19 spread. The virus could be transported by the PM, but it could be partially or completely unable to spread the disease. Several authors have found the presence of SARS-CoV-2 RNA in PM collected in urban areas during epidemic waves, but it was completely inhibited due to its low resistance in the presence of adequate solar radiation. However, this behavior is extremely complicated because all variables remain unclear. For instance, some authors have found different rates of inhibition depending not on the point of sampling but on the original source, and therefore, the composition of the PM.

Regarding numerical and statistical analyses, the acute role of PM alone in the spread of COVID-19 can be assumed to be negligible. However, further studies are needed, especially on the role of environmental conditions in influencing SARS-CoV-2 inactivation. Clear and complete knowledge about the variables affecting COVID-19 spread, including the impact of PM, will be helpful to prevent and/or limit the spread of future diseases. 

## 5. Conclusions

This work discusses the latest findings about the possible pattern between PM and COVID-19. For PM, the current literature suggests two possible mechanisms of action in SARS-CoV-2 spread: (i) a chronic and (ii) an acute role. The literature results have proven that short-term and exposure to PM at high concentrations can negatively affect the respiratory system, while long-term exposure can also have other additional effects on human health, such as on the cardiovascular system. Moreover, some authors have also suggested the possible role of PM in altering the expression of ACE2, which also acts as a receptor for SARS-CoV-2. These aspects leave exposed people more susceptible to severe forms of COVID-19 symptoms, including death. Some authors have also linked previous exposure to high concentrations of PM with a higher number of infections, probably due to the broad swab testing caused by a lower number of asymptomatic cases. However, this aspect should be further studied. Although the results from the scientific community regarding the presence of SARS-CoV-2 in PM are conflicting, the influence of particulate alone in promoting infections due to the transport of SARS-CoV-2 can be considered negligible based on several numerical and statistical analyses. In the opinion of the authors, more efforts should be made to evaluate the influence of environmental conditions on the possible inactivation of the virus, especially when transported on PM. Complete knowledge about the environmental variables that influence COVID-19 morbidity and mortality and the spread of SARS-CoV-2 represents a key step toward better preventing and managing the spread of future diseases.

## Data Availability

All data generated or analyzed during this study are included in this published article.
